# COVID-19 impact and virtual medical education

**DOI:** 10.30476/jamp.2020.86070.1213

**Published:** 2020-07

**Authors:** SHIMA TABATABAI

**Affiliations:** 1 Medical Education Group, Medical Ethics and Law Research Center, Shahid Beheshti University of Medical Sciences, Tehran, Iran

**Keywords:** COVID-19, Pandemics, Virtual education, Medical education, Simulation

## Abstract

The Corona-Virus Disease 2019 (COVID-19) Pandemic has had a tremendous effect on medical education. It is also challenging the medical educationists' ability to adapt to this whole unique situation. Considering the hospital-based education, clinical mentors, and students in all health professions are potential carriers.

However, the current crisis is revitalizing the necessity for online learning opportunities and virtual education. Most medical schools are following reacting to lockdown with a shift to live online or video-based learning. Maintaining standard in medical education, keeping the clinical learning on stream, and minimizing the assessment disruption are unprecedented challenges under pandemic conditions. Adaptation to this new situation is necessary to prepare future clinicians for practice.

This commentary discusses how this pandemic may affect medical education. In this commentary, the author highlights the importance of virtual education and the potential implications of integrating virtual simulation technologies into medical education for the future of clinical competency learning and assessment.

## Introduction

The Corona-Virus Disease 2019 (COVID-19( outbreak, as a rapidly evolving situation, has influenced all members of the community and has caused tremendous disruption in all life aspects, not least in the health professions who provide front-line care for patients. Currently, the ongoing pandemic has a tremendous effect on medical education. It is also challenging medical educationists' ability to adapt to this whole unique situation. Considering the hospital-based education, clinical mentors, and students in all health professions, frequently working in the most difficult circumstances are potential victims and carriers for the virus ( 1 ).

In the state of emergency, Medical educators as role models are facing major challenges to train as the next generation of doctors ( 2 ). We have to respond to the immediate concerns among medical students, to channel our student’s energy from panic to resolve this problem and to apply our energy to what we know we can do about medical education based on the lessons learned during pandemics. 

Universities across the world have reacted quickly to the crisis, having announced immediate closures. Medical Schools are enacting emergency contingencies that allow their student populations to complete the semester remotely ( 3 ). In addition, the current ongoing crisis is revitalizing the necessity for virtual learning opportunities. Most medical schools are preparing to transform the medical education from real classes to the virtual education to minimize the teaching and assessment disruption ( 4 ). The transformative drivers are associated with the pandemic length of time. They are likely to interact with other factors such as types of available resources and emergent technology.

### *How to transform to the virtual education?*

Virtual education refers to instruction in a learning environment where educators and students are separated by time or space, or both, and the instructors provide course content through course management applications, multimedia resources, the Internet, videoconferencing, etc. Students receive the content and communicate with the teacher via the same technologies ( 5 ). 

 Since the perspective of medical education after the COVID-19 pandemic will transform ( 1 ), it is necessary to transform the medical education through the following suggested steps:

**1. Determining medical education priorities during the pandemic:** As the COVID-19 outbreak has rapidly transitioned, following the social distancing practices, keeping learners safe, supported and engaged, and helping keep education programs moving forward with high quality, are the medical education systems’ priorities. 

**2. Assessing available resources:** Digital learning management systems and massive open online courses offer free online courses and classes aimed at the professional development of educators. In addition, there are also many free tools enabling video communications, video and audio conferencing, chats, and webinars for medical educators such as Zoom, Skype, and Google Hangouts Meet ( 2 , 4 ). These online communication tools support collaboration and the sharing of expertise between universities and providers. 

In Iran, most universities of medical sciences have created a platform for the provision of virtual education. Regarding authentic medical education, Iran’s Virtual University of Medical Sciences (VUMS) has provided resources and educational content to help medical schools all around the country. At VUMS, the national learning management system was established to provide an electronic platform for the medical sciences education. This learning management system (LMS), like comparable LMSs, was intended to help instructors reflect on medical sciences students’ processes of learning as well as meeting the needs of individual medical sciences students. Some of its major facilities include course content repository, management of users, courses, instructors, and facilities, and generation of reports, course calendar, learning path, and discussion forum where members could provide their own contributions, prepare exercises, assign deadlines to and correct them, display scores and transcripts, etc. Besides, the national virtual university of medical sciences is providing what are known as MOOCs - massive open online courses- in partnership with leading universities of medical sciences in Iran. Under the current circumstances, the educational contents are produced by the professors of different universities to provide integrated authentic learning experiences for Iranian medical students.

**3. Determining challenges and related solutions:** This outbreak widens the gap between those able to access online learning opportunities and those who are not. Many Students suffer a type of unequal access whereby they lack the digital connections ( 6 ). Maintaining performance standards and quality assurance are unprecedented challenges under pandemic conditions.

 As the COVID-19 pandemic continues to develop, it could decrease the medical students’ and residents’ exposure to specific clinical conditions, and causes a significant effect on their clinical clerkship evaluation and competency assessment exams ( 1 , 2 ). Keeping the students engaged and on track with curriculum-driven coursework, even though they’re learning remotely, are unprecedented challenges under pandemic conditions. HD-quality mobile or fixed-camera live streaming to establish a standardized, classroom-like experience in real-time improves interaction and ensures that the remote learners can participate at the same baseline ( 4 , 7 ). 

**4. Prognosis:** The latest innovations in flexible educational technologies will change the future of medical education ( 7 ). Providing engaged and high-quality medical education will be guaranteed through virtual medical universities and simulation-based solutions for healthcare training environments ranging from clinical simulation management software and hardware to counselor education, case development, and virtual patient training ( 6 , 8 ). Virtual learning and education require careful thinking about how educators are equipped for the shift. 

**5. Preparing for future transformation after the pandemic:** The long-term impact of the current pandemic on medical education could be considerable. To maintain high-quality medical education, administrators and educators forced to look for innovative technologies. Medical Educationists will need to use emergent technologies which impact on the future way that their institutions will provide medical education ( 9 ). 

### *Integrating virtual reality and simulated clinical experiences into medical education*

Virtual reality is the use of software to create a simulated environment where educators can engage in virtual situations in a way that feels real ( 10 ). 

Simulation is a vital part of medical education and students just don’t get to do it enough. Virtual reality simulation (VRS) has the unique ability to make learners believe they are in a different environment. This allows medical students to learn from virtual simulated clinical experiences as they would do in hospital-based real experiences. Virtual simulation-based medical education has been found to be ‘superior to traditional clinical education ( 10 , 11 ). Integrating VRS into Medical education has enabled medical educationists in providing the simulated clinical experiences to a greater number of medical students in a shorter time. 

VRS is used in postgraduate medical education by the University of Northampton and Oxford University Hospitals. VRSs have also been used across England’s health education and healthcare systems since 2019 ( 10 , 11 ).

The future of medical education lies in technological developments and the ongoing integration of virtual clinical simulation experiences into medical curriculum. As pressure to increase delivery of simulation continues, VR simulation will continue to expand ( 12 - 14 ). A number of institutions are also investigating VRS from the standpoint of objective structured clinical examinations (OSCES), as a method of decreasing the cost and increasing the objectivity of their assessment processes ( 15 ).

### *How Virtual reality simulation could adapt the clinical education and assessment with the impact of COVID19*

It is essential to ensure medical students’ safety and on-time evaluation during this pandemic crisis. The Universities of Medical Sciences need to invest in individualized learning for competency-based education and in the technologies necessary for virtual assessments of student clinical competency ( 4 , 8 ). 

Virtual simulation-based education products provide the necessary tools to prepare benchmarking and best practice insights to medical students, better preparing them for real-world practice and these tools are set to expand over the coming years ( 8 , 12 , 13 ). Simulation-based educational technologies can be effective for the transformation of clinical education, even the most complicate traditional-to-virtual universities, in the following ways:

The simulation-based educational technologies allows medical education to quickly move to a virtual environment ( 13 , 14 ). These technologies schedule simulated meetings, facilitate virtual education with video conferencing and reviewing clinical decision making through simulated clinical experiences.

The VRS changes traditionally time-consuming manual processes to create an integrated approach that improves clinical outcomes, and saves time. The VRS platforms allow learners to access the clinical scenarios via the computer or the mobile device regardless of their location, improve educational task flows, and customizes remote learning parameters specific to the program requirements.

By simulation-based platform, clinical educators could conduct high-impact, case-based simulated training scenarios online and run live, virtual Objective Structured Clinical Examinations (OSCEs) from anywhere ( 12 , 15 ). Simulation-based Virtual OSCE could offer the perfect cloud-based platform to lead standardized patient confronts in a completely virtual, video-enabled environment to address all clinical education needs ( 14 , 15 ). Therefore, the virtual simulation platform helps medical educationists to maximize the learning potential of OSCEs. In addition, Simulation-based Virtual OSCE facilitates fostering core competency for clinical decision making to transform clinical practice into telemedicine forms in the context of virtual medical education. 

## Conclusion

We have to adapt to the new situation to prepare future clinicians for practice under pandemic conditions. Educational methods are changing and new domains of medical sciences and technological innovations, as well as educational technologies, are growing. Today medical sciences students are digital natives and rely more and more on virtual education and virtual reality simulated experiences. Virtual reality simulation products are the bases for simulation-based virtual education that are urgently needed by many medical education programs. Simulation-based virtual education has the potential to ensure that transformative developments continue to benefit medical education across the world. We need new innovative strategies for utilizing the medical education system to support uninterrupted education and assessment. Considering the benefits of virtual reality and simulation-based technologies, medical university administrators should invest in simulation-based virtual educational products to keep the clinical competency education and assessment on stream.
